# Dengzhanxixin Injection Ameliorates Cognitive Impairment Through a Neuroprotective Mechanism Based on Mitochondrial Preservation in Patients With Acute Ischemic Stroke

**DOI:** 10.3389/fphar.2021.712436

**Published:** 2021-08-30

**Authors:** Haiting An, Wuhai Tao, Ying Liang, Peng Li, Min Li, Xiaxia Zhang, Kewei Chen, Dongfeng Wei, Daojun Xie, Zhanjun Zhang

**Affiliations:** ^1^ State Key Laboratory of Cognitive Neuroscience and Learning & IDG/McGovern Institute for Brain Research, Beijing Normal University, Beijing, China; ^2^ BABRI Centre, Beijing Normal University, Beijing, China; ^3^ Center for Brain Disorders and Cognitive Sciences, Shenzhen University, Shenzhen, China; ^4^ School of Biomedical Engineering, Capital Medical University, Beijing, China; ^5^ Institute of Basic Medicine Research, Xi Yuan Hospital affiliated to China Academy of Chinese Medical Sciences, Beijing, China; ^6^ Banner Good Samaritan PET Center, Banner Alzheimer’s Institute, Phoenix, AZ, United States; ^7^ Institute of Basic Research in Clinical Medicine, China Academy of Chinese Medical Sciences, Beijing, China; ^8^ The First Affiliated Hospital of Anhui University of Chinese Medicine, Hefei, China

**Keywords:** Dengzhanxixin injection, acute ischemic stroke, cognitive impairment, cerebral grey matter, mitochondria

## Abstract

Acute ischemic stroke (AIS) is a global health burden and cognitive impairment is one of its most serious complication. Adequate interventions for AIS may have the potential to improve cognitive outcomes. In the present study, we selected *Erigeron breviscapus* (Vaniot) Hand.-Mazz. injection (Dengzhanxixin injection, DZXI), a widely used Chinese herbal injection, in contrast to edaravone as the positive control drug to test its potential to ameliorates neurological and cognitive impairments caused by AIS. We performed a 2-week randomized trial with these two drugs in AIS patients presenting mild to moderate cognitive impairments. Neuropsychological tests and MRI examinations showed that DZXI attenuated the neurological and cognitive impairments of patients and protected the grey matter in specific regions from ischemic damage. Notably, DZXI exerted better effects than edaravone in some neuropsychological tests, probably due to the protective effect of DZXI on grey matter. To explore the therapeutic mechanisms, we carried out an experiment with a middle cerebral artery occlusion rat model. We found that DZXI decreased the infarct volume and increased the survival of neuronal cells in the ischemic penumbra; furthermore, DZXI modulated the mitochondrial respiratory chain process and preserved the mitochondrial structure in the brain tissue. Overall, our data suggested that the administration of DZXI is effective at ameliorating neurological and cognitive impairments in AIS, and the underlying mechanisms are related to the protective effects of DZXI on cerebral neurons and neuronal mitochondria.

## Introduction

Globally, stroke is one of the leading causes of death and disability with yet rapid increase (GBD, 2019). In China, more than two million new cases of stroke occur annually, and stroke is also associated with the highest death and disability-adjusted life-years lost among all diseases (Wu et al., 2019). Being the overwhelming majority, ischemic stroke accounts for approximately 70% of all incident stroke cases according to a China nation-wide community study (Zhou et al., 2019). Certain infarctions in critically important brain regions may directly cause cognitive impairment in patients with acute ischemic stroke (AIS) (Wang et al., 2021). In fact, cognitive impairment is one of the most common neurological sequelae of ischemic stroke, bitter suffering for patients, as well as a heavy burden for families and the society (Howard, 2016). It affects up to one third of stroke survivors and the exact prevalence could be much higher because of selection bias, issues on feasibility of cognitive testing, and attrition to follow-up in studies (Pendlebury and Rothwell, 2009; Pendlebury et al., 2015a; Pendlebury et al., 2015b). Not surprisingly, the daily activities of patients with AIS are seriously affected.

In addition to the abnormal cognitive function, the mortality after stroke and is closely related to the stroke severity in AIS (Zietemann et al., 2018). Finally, recent results also suggested that the greatest long-term risk of dementia might occur in patients with severe stroke (Dichgans, 2019). These indicate that stroke is not just an associate, but can be an initiator of cognitive deterioration (Sagnier and Sibon, 2019). Ischemia triggers a cascade of events in the brain, including impediment of aerobic energy metabolism, extensive generation of reactive oxygen spices, and mitochondrial injury among others (George and Steinberg, 2015). These events harm neurons directly and also disrupt the blood brain barrier causing secondary damages. The final outcome is the drastic loss of neurons, which is macroscopically manifested as the formation of severe lacuna and atrophy of the brain grey matter (Sekerdag et al., 2018). At present, thrombolytic therapy for AIS with i.v. recombinant tissue-plasminogen activator (rt-PA) and endovascular thrombectomy using a stent retriever are the major effective treatments. To date, there is limited evidence for specific therapeutic strategies for preventing cognitive impairment in AIS.

**FIGURE 1:**
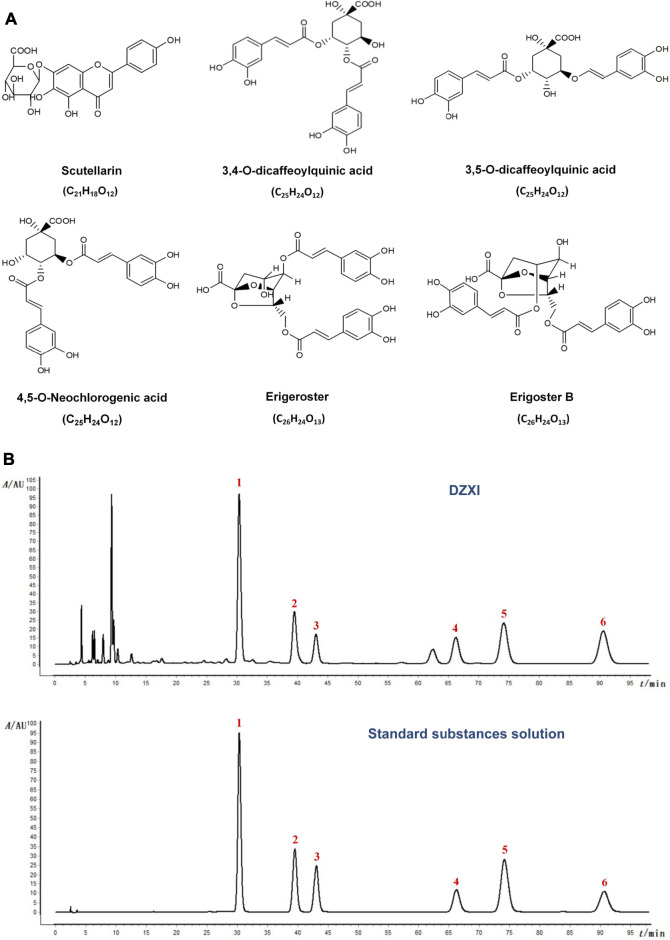
**(A)** The chemical structure and molecular formula of active compounds in DZXI. **(B)** Chromatogram of HPLC analysis of DZXI and standard substances solution. The active compounds: 1. scutellarin; 2. 3,4-O-dicaffeoylquinic acid; 3. 3,5-O- dicaffeoylquinic acid; 4. erigoster B; 5. 4,5-O-dicaffeoylquinic acid; 6. erigeroster.

*Erigeron breviscapus* (Vaniot) Hand.-Mazz. (Dengzhanxixin), a Chinese medicinal plant mainly grown in southwest China, has a long history of medicinal use in Chinese medicine. Its various preparations have been extensively used in clinics in China to treat ischemic cardio-cerebral vascular diseases for a long time (Chai et al., 2013). Chinese herbal injections are prepared by extracting and purifying effective substances from herbs (or decoction pieces) using modern scientific techniques and methods. As a widely used Chinese herbal injection, *Erigeron breviscapus* (Vaniot) Hand.-Mazz. injection (Dengzhanxixin injection, DZXI) were phenolic acid extract from *Erigeron breviscapus* (Vaniot) Hand.-Mazz. and has been officially listed in the Chinese pharmacopoeia (National Pharmacopoeia Commission, 2020). It has been approved by China Food and Drug Administration (CFDA, with its approval number Z53021569) (Wang J. et al., 2017). DZXI has been widely used for AIS and cardiovascular diseases, and thousands of clinical trials showed its benefits for AIS (Li et al., 2017). The active compounds of DZXI include scutellarin, 3,4-O-dicaffeoylquinic acid, 3,5-O-dicaffeoylquinic acid, erigoster B, 4,5-O-dicaffeoylquinic acid and erigeroster (Lin, 2020). The chemical structure and molecular formula of these active compounds are shown in Figure 1A. Seen from its medical use instruction, DZXI can activate blood circulation to dissipate blood stasis and relieve pain. According to previous studies, its therapeutic mechanisms include anti-oxidation, anti-inflammation, and neuroprotection (Li et al., 2017). These characteristics suggest that DZXI has a great potential to rescue brain acute ischemia injury and ameliorate neurological and cognitive impairments in AIS patients.

In the present study, we investigated the efficacy of the DZXI intervention for cognitive impairments in AIS patients and explored the possible mechanism in rats with middle cerebral artery occlusion (MCAO), the model commonly employed in pre-clinical ischemic stroke studies (McCabe et al., 2018). We also used edaravone as positive control drug, which is a well-known neuroprotective drug that prevents brain damage by capturing and reducing excessive reactive oxygen species (ROS) (Matsumoto et al., 2018). In the clinical investigation, AIS patients presenting mild to moderate cognitive impairments were recruited to a 2-weeks trial. We used a battery of neuropsychological tests to investigate the cognitive function, and used MRI to examine the grey matter. In the experimental investigation, we further examined the underlying therapeutic mechanisms in the rat model with neuroimaging, neurohistological examinations and a proteomic assay. This study may be significant for searching effective drugs of AIS, and the therapy of vascular cognitive impairment.

## Materials and Methods

### Drugs and Chemical Analysis

#### Drugs, Chemicals, and Reagents

Dengzhanxixin injection (specifications: 5.32 mg scutellarin, 2.26 mg 3,4-O-dicaffeoylquinic acid, 1.10 mg 3,5-O-dicaffeoylquinic acid, 1.79 mg erigoster B, 2.70 mg 4,5-O-dicaffeoylquinic acid, and 11.26 mg erigeroster, 10 ml/ampoule, Lot No. 20180137) were provided by Yunnan Biovalley Pharmaceutical Co., Ltd (Kunming, China). Edaravone (specifications: 30 mg, 20 ml/ampoule, Lot No, 80-160609) were provided by Simcere Corporation (Nanjing, China). Scutellarin (Lot No, 110842-201709; purity 91.7%), 3,5-O-dicaffeoylquinic acid (Lot No, 111782-201807, purity 94.3%), and 4,5-O-dicaffeoylquinic acid (Lot No, 111894-201102, purity 94.1%) were purchased from the National Institute for Food and Drug Control (Beijing, China). 3,4-O-dicaffeoylquinic acid (Lot No, 3089, purity 98.0%) was purchased from Shanghai Bainian Standand Testing Technology Co., Ltd. Erigoster B (Lot No, 20190701, purity 92.4%) and erigeroster (Lot No, 20190701, purity 93.3%) were provided by Yunnan Biovalley Pharmaceutical Co., Ltd (Kunming, China) and Chengdu Pusi Biotechnology Co., Ltd (Chengdu, China). Methanol and acetonitrile of HPLC grade were obtained from Merck (LiChrosolv, Merck, Darmstadt, Germany). Trifluoroacetic acid of HPLC grade was obtained from Tedia Company, Inc (Fairfield, OH, United States).

#### Preparation Protocol of Dengzhanxixin Injection

*Erigeron breviscapus* (Vaniot) Hand.-Mazz. were decocted twice with water, 2 h per time. The decoction were combined and filtered. The filtrate was condensed into extractum with a relative density of 1.15∼1.25 (75°C). The extractum were diluted with three times volume of water and filtered after adding 5% sodium hydroxide solution to adjust the pH value to 7.5∼8.5. The filtrate was added 10% sulfuric acid solution to adjust the pH value to 2∼3 and filtered to get the filtrate and precipitation. The precipitation was dissolved with equal amount of water. The 10% sodium hydroxide solution was added to adjust the pH value to 5∼6 and the precipitation solution was filtered. The filtrate was added 20% sulfuric acid solution to adjust the pH value to 1∼2 and filtered. The precipitation was washed with 90% ethanol for 4 times and dissolved with appropriate amount of 65% ethanol. The precipitation solution was added 0.5% sodium hydroxide solution to adjust the pH value to 5∼6 and filtrated. The filtrate was added 10% hydrochloric acid solution to adjust the pH value to 1∼2 and filtered. The precipitate was washed four times with 90% ethanol and dried to powder in vacuum condition. The filtrate passed through a polyamide column and the polyamide column was eluted with four times volume water, four times volume of 40% ethanol and 2 times volume of 70% ethanol, respectively. The water eluent was discard. The eluent of 40 and 70% ethanol were collected and concentrated to the relative density of 1.03∼1.08 (70°C). The eluent was extracted with ethyl acetate for two times. The ethyl acetate extract was concentrated to a relative density of 1.20∼1.30 (45°C) after recycling ethyl acetate solution. Water for injection was added to powder and ethyl acetate extract, respectively and 5% sodium hydroxide was added to adjust pH to 7.5∼8.5, and then filtered. The sodium chloride (8 g) and activated carbon (0.2 g) were dissolved in water for injection and the mixed solution were boiled and filtered. The powder filtrate, ethyl acetate extract filtrate and sodium chloride filtrate were combined and mixed. The above mixed filtrate was added water for injection to 1,000 ml, then filtered and sterilized (National Pharmacopoeia Commission, 2020).

#### Instrument and Quantitative Chemical Analysis of Active Compounds

A high-performance liquid chromatography (HPLC) method was carried out using the Waters Alliance HPLC System equipped with e2695 separation unit, 2998 diode-array detector, and empower chromatographic work station (Waters, United States). XS3DU micro balance (Mettler Toled, Switzerland). Preparation of DZXI sample as follows: 2 ml DZXI was placed into a 10 ml volumetric flask, adding 0.7 mmol/l ethylenediamine tetraacetic acid disodium solution to the scale. The mixed solution were filtered with 0.45 μm membrane after shaken well. Preparation of standard solutions as follows: six reference substances including scutellarin, 3,4-O-dicaffeoylquinic acid, 3,5-O-dicaffeoylquinic acid, erigoster B, 4,5-O-dicaffeoylquinic acid, and erigeroster were accurately weighed. Methanol was added to prepare their mixed solution and their individual concentrations were as follows: 60, 60, 40, 60, 70, 100 μg/ml. The mixed solution were filtered with 0.45 μm membrane after shaken well.

Analytical conditions: Waters Sunfire C18 chromatographic column (250 mm × 4.6 mm, 5 μm, Octadecyl silane bonded silica gel filler) with [A(methanol: acetonitrile 30:70)-B(0.1% trifluoroacetic acid solution)] (18:82) as mobile phase, flow rates1.0 ml/min, column temperature 40°C. The detection wavelength of scutellarin and dicaffeoylquinic acid were set at 335, 327 nm, respectively. 3,5-O-Dicaffeoylquinic acid was chosen as the internal reference substance, the relative correction factors of 3,4-O-dicaffeoylquinic acid, erigeron B, 4,5-O-dicaffeoylquinic acid, and erigeroster were determined by multi-point correction, and the relative retention time of the chromatographic peaks of the compounds to 3,5-O-dicaffeoylquinic acid were calculated, respectively.

### Clinical Trial

#### Study Design

This clinical trial was registered in Chinese Clinical Trial Registry (URL: http://www.chictr.org.cn/index.aspx, registration number: ChiCTR-IPR-17011051), and the study content was approved by the Medical Ethics Committee of the First Affiliated Hospital of Anhui University of Chinese Medicine with approval number 2016AH-16. We provided all participants and their families with detailed information regarding the methods and purpose of this study, and all patients signed the informed consent form.

#### Patients

The trial inclusion criteria for patients were as follows: 1) meeting the diagnostic criteria for AIS in 2014; 2) having a National Institutes of Health Stroke Scale (NIHSS) score between 3 and 10 points; 3) having a Hachinski Ischemic Scale (HIS) score ≥4 points; 4) having a Mini-Mental Status Examination (MMSE) score between 11 and 26 points; 5) being in the ages ranges of 35–80 years old; 6) voluntarily participating in this trial and signing the informed consent form; and 7) having a certain level of education that enabled the patient to read simple articles and write simple sentences.

Patients were excluded for any of the following reasons: 1) being in recovery period or sequelae of cerebral infarction, or being diagnosed as transient ischemic attack; 2) being diagnosed as cerebral hemorrhage or subarachnoid hemorrhage; 3) being diagnosed as primary dementia accompanied by another cognitive impairment; 4) suffering from stroke caused by brain tumors, brain trauma, etc.; 5) suffering from cerebral embolism due to rheumatic heart disease, coronary heart disease and other heart disease combined with atrial fibrillation; 6) being with other serious diseases, such as diseases of the liver, kidney, and osteoarthritis; 7) suffering from mental disorders or severe dementia; 8) being unsuited to MRI examination; 9) being allergic to the test drug; and 10) participating in other clinical drug trials.

The criteria of ceasing trial for patients were as follows: 1) having serious adverse events occurred during the study; 2) displaying poor compliance with the study protocol; 3) withdrawing the informed consent by the patient his/herself or by his/her family members; 4) being considered by the investigators that withdrawal from the study is the best choice for the patient for reasons of efficacy and safety; and 5) seriously violating the research protocol.

The patients were randomly allocated to DZXI group and edaravone group. Neither the patients nor investigators were aware of the allocation information until the study was complete. During the clinical trial period, patients in the treatment groups received 97.72 mg DZXI once a day or 30 mg edaravone twice a day, intravenously guttae, combining with routine Western medicine treatment, for 2 weeks. All patients received a battery of neurological deficit tests and neuropsychological tests at both the beginning and ending of the study (Table 1). The flowchart of procedure of the clinical trial was shown in Supplementary Figure S1.

**TABLE 1:** Demographics and neuropsychological test scores (Mean ± SD).

	DZXI (*n* = 52)		Edaravone (*n* = 31)		Group effect		Time effect		Interaction effect	
	Baseline	2 weeks	Baseline	2 weeks	*F*	*P*	*F*	*P*	*F*	*P*
**Characteristics**										
Age (y)	61.98 ± 10.09		60.45 ± 9.18							
Gender (M/F)	32/20		22/9							
Education (y)	8.19 ± 3.49		7.27 ± 3.79							
**Neurological deficits**										
NIHSS	5.02 ± 2.07	3.58 ± 1.88^a^	5.57 ± 2.50	3.17 ± 2.12^a^	0.002	0.967	2.157	0.146	7.662	0.007^**^
mRankin	2.98 ± 0.90	2.37 ± 1.09^a^	2.47 ± 1.04^b^	1.73 ± 0.94^a^	7.301	0.008	0.014	0.906	1.196	0.278
ADL	37.67 ± 11.87	32.08 ± 11.46^a^	31.70 ± 9.53^b^	26.40 ± 8.33^a^	6.218	0.015	0.000	0.982	0.000	0.998
**General Mental Status**										
MMSE	22.06 ± 5.12	23.78 ± 5.46^a^	23.21 ± 3.30	25.28 ± 3.36^a^	2.539	0.115	2.776	0.100	0.144	0.705
**Executive function**										
SCWT B (time)	64.41 ± 21.74	56.08 ± 19.37^a^	88.56 ± 21.54^b^	77.40 ± 19.33^a^	20.500	0.000	0.070	0.792	1.884	0.174
SCWT B (N)	43.45 ± 5.08	44.12 ± 6.42^a^	40.72 ± 4.00	42.08 ± 3.32^a^	2.765	0.101	2.068	0.155	0.760	0.386
SCWT C (time)	169.56 ± 58.34	152.36 ± 49.51^a^	172.00 ± 43.04	145.24 ± 33.17^a^	0.330	0.567	5.268	0.025	1.341	0.251
SCWT C (N)	39.86 ± 4.81	41.18 ± 6.63^a^	37.40 ± 4.72^b^	39.40 ± 3.56^a^	4.321	0.041	0.494	0.484	0.190	0.664
**Attention**										
TMT A	113.94 ± 54.02	95.19 ± 43.62^a^	89.89 ± 46.21^b^	69.30 ± 35.28^a^	6.127	0.016	0.997	0.321	0.115	0.736
**Visuospatial ability**										
CDT	17.95 ± 7.97	24.46 ± 5.93^a^	21.19 ± 4.89^b^	23.27 ± 4.44^a^	0.770	0.384	0.166	0.685	7.969	0.006^**^
**Episodic memory**										
AVLT(N1-N5)	12.92 ± 6.68	15.84 ± 7.01^a^	20.70 ± 7.01^b^	22.93 ± 6.57^a^	29.025	0.000	7.743	0.007	1.002	0.320
AVLT (delay recall)	15.70 ± 5.24	18.86 ± 4.31^a^	12.28 ± 2.31^b^	11.79 ± 3.96	29.395	0.000	0.347	0.558	24.901	0.000^**^
**Working memory**										
DST (forward)	5.75 ± 1.06	6.61 ± 1.02^a^	5.83 ± 1.49	6.67 ± 1.56^a^	0.143	0.706	0.424	0.517	0.003	0.956
DST (backward)	2.75 ± 0.63	3.10 ± 0.54^a^	3.07 ± 1.08	3.70 ± 1.18^a^	7.742	0.007	4.887	0.030	3.897	0.052
**Language ability**										
CVFT (fruit)	18.90 ± 13.49	20.80 ± 14.31^a^	20.80 ± 14.31^b^	11.93 ± 1.86^a^	10.302	0.002	6.781	0.011	1.583	0.212
CVFT (vegetable)	18.49 ± 11.96	20.86 ± 13.12^a^	10.97 ± 1.88^b^	12.13 ± 1.70^a^	12.524	0.001	0.088	0.768	0.994	0.322

The comparisons of the neuropsychological scores between the two groups (DZXI and edaravone) were performed with two-way repeated measures ANOVA, after controlling age, gender, and education status as covariates. ^∗∗^ *p* < 0.001. Post hoc pairwise comparisons were performed using t-test,

a Significantly different, between the two time points in DZXI or edaravone group.

b Significantly different, between the DZXI and edaravone group at baseline.

NIHSS, National Institutes of Health Stroke Scale; mRS, modified Rankin Scale; ADL, activities of daily living; MMSE, Mini-Mental State Examination; SCWT, Stroop colour-word test; TMT, trail making test; AVLT, auditory verbal learning test; CDT, clock-drawing test; DST, digit span test; CVFT, category verbal fluency test.

#### MRI Examination and Grey Matter Preprocessing in Human Subjects

MRI data were acquired from patients on a 3.0 T Siemens scanner at the First Affiliated Hospital of Anhui University of Chinese Medicine. The images were acquired using the following parameters: T1: 3T MRI; TR = 8.16 ms; TE = 3.18 ms; TI = 450; flip angle = 12°; field of view = 256 × 256 mm^2^; matrix size = 256 × 256; slice thickness = 1 mm; voxel size = 1 × 1 × 1 mm³; and slices = 256.

We used SPM12 software and the CAT12-toolbox to preprocess the T1-weighted structural images. The images were first converted from the DICOM (Digital Imaging and Communications in Medicine) format to NIfTI (Neuroimaging Informatics Technology Initiative) format. Then, the images were segmented into grey matter, white matter, and cerebrospinal fluid with the default setting. The total intracranial volume was estimated and used to correct for different head sizes and volumes in the next step. The GM images were then mapped to MNI-152 standard space with an isotropic voxel resolution of 1.5 mm × 1.5 mm × 1.5 mm. We used the “check sample homogeneity” module in the CAT12 toolbox to identify outliers by visualizing the correlation between the volumes to control the images quality, and those subjects with obvious image problems were excluded. Finally, the modulated GM volume images were smoothed with a Gaussian kernel of 8 mm full-width-half- maximum (FWHW).

### Animal Experiment

#### Animals and Grouping

The animal experiments were conducted under the direction of NIH Guidelines for the Care and Use of Laboratory Animals (NIH Publications No. 80-23, revised 1996). The procedures were approved by the Animal Care and Use Committees of Beijing Normal University, China. Adult male Sprague-Dawley rats (270 ± 10 g) were purchased from Beijing Vital River Laboratory Animal Technology Co., Ltd. The animals were allowed to acclimate for 7 days before the experiments. Rats were randomly divided into a sham-operated (sham) group, model group, DZXI-treated (DZXI) group, and edaravone-treated group (*n* = 15 rats each group). DZXI (8.8 mg/kg) or edaravone (2.7 mg/kg) was intravenously injected into rats, the first drug treatments were performed immediately after the middle cerebral artery occlusion (MCAO) surgery, the subsequent ones were continually performed with the interval of 12 h, i.e., twice a day; and the drug treatments lasted for 7 days. The rats in the sham group and the model group were treated with the same volume of saline in the same way.

#### Middle Cerebral Artery Occlusion Surgery

Before surgery, rats were fasted overnight, with free access to water. Animals were anaesthetized with 3.5% chloral hydrate (350 mg/kg body weight, i.p.) and MCAO was performed. Briefly, the right common carotid artery, external carotid and internal carotid were exposed through a midline cut. A nylon monofilament coated at the tip with 5 mm of silicone (diameter 0.36 ± 0.02 mm) was inserted into the external carotid artery, and was pulled to the internal carotid artery to occlude the origin of the middle cerebral artery. Model animals were subject to 1.5 h of MCAO, and then to reperfusion achieved by a gentle withdrawal of the occluding thread. The rectal temperature was maintained at 37 ± 0.5°C with a thermostatically controlled heating blanket throughout the surgical procedure. Sham-operated animals were subjected to the same procedure, except for the insertion of the nylon monofilament.

#### Brain MRI Examination in Rats

At 24th h and 7th day after drugs treatment, the rats were subjected to MRI scan. The animals were anaesthetized with isoflurane (mixed in pure O_2_ with concentrations of 1.5–2%) which was delivered *via* a nose cone; and the animals were under with continuous monitoring of the heart rate and respiration during the imaging sessions. The MRI experiments were performed with a 7 T/20-cm- diameter bore Bruker Biospec scanner at the Center for Medical Experiments and Testing, Capital Medical University. A volume coil was used for radiofrequency transmission, and a quadrature surface coil was used for signal detection. T2-weighted images were acquired with the following RARE sequence: TR = 6,300 ms, TE = 24 ms, matrix size 256 × 256, and scanning layer thickness = 0.3 mm. The imaged FOV included the brain olfactory bulb, the cerebellum and extra-brain tissue.

The original rat MRI data were converted into an analyze format image, and the voxel size was enlarged 10 times during the conversion, to be convenient for other subsequent analysis software. In MRIcro, the skull and other non-brain tissues in the image were manually removed. Then, the SPM DARTEL toolkit was used for the following preprocessing steps: 1) segmenting all the original structural images to obtain the grey matter image of each rat, and performing field non-uniformity correction to reduce the linear grey scale of the image caused by uneven surface coils; 2) separately preparing the grey matter and white matter templates by using DARTEL; 3) using the generated grey matter templates to spatially normalize the grey matter images of all rats; and 4) smoothing the normalized grey matter images using Gaussian kernel functions.

The hyperintense regions on T2-weighted MR images were recognized as infarcts, and each individual embolic infarct was manually traced on individual MRI slices using MRIcron neuroimaging software. Then, the volume of each infarct as well as their combined volume was calculated with incorporated slice thickness and inter-slice gap. Infarct distribution was analyzed using SPM (Zhou et al., 2017).

#### Nissl Staining and Haematoxylin and Eosinmiddle cerebral artery occlusion Staining

After the rats were anesthetized with 3.5% chloral hydrate, their brains were perfused with warm saline *via* the left ventricle and were fixed with 2% glutaraldehyde and 4% paraformaldehyde (PFA). Then the tissues were embedded in paraffin and serially coronally sectioned. The sections were dewaxed with xylene, dehydrated with a gradient of alcohol solutions, and washed with running water. In HE staining, the brain sections were stained with haematoxylin and differentiated with 0.5% hydrochloric acid alcoholic solution, followed by washes with running water. Then, sections were returned to a blue colour by incubating them with a saturated lithium carbonate solution for 1 min and stained with a 0.1–0.5% eosin solution for 10 min. In Nissl staining, the brain sections were stained with 0.5% (m/v) toluidine blue for 15 min. The sections were observed with a microscope and photographed. Eight consecutive slices of each rat and one scope of each slice were imaged and counted. The areas and the numbers of Nissl bodies and surviving neural cells in sections of six rats of each group were measured by Image-Pro plus 6.0. The value was normalized to the total hippocampus or cortex area in the images. Averages of each rat were calculated across images and each rat contributed a single value (Wang et al., 2014).

#### Transmission Electron Microscopy

The tissues around ischemic core were dissected and cut into slices. Subsequently, the specimens were fixed with 2.5% glutaraldehyde for 180 min, rinsed with 0.1 M phosphate buffer three times, fixed with 1% osmium tetroxide, dehydrated with a graded series of ethanol solutions, embedded in araldite, polymerized for 2 days, and then cut into ultrathin sections. Finally, sections were stained with uranyl acetate and lead citrate and the neuron ultrastructure in penumbra regions were observed using a transmission electron microscopy (TEM).

#### Proteomic Analysis

In the model and the DZXI groups, tissues (four replicates in each group) around the ischemic core were harvested after 7 days of treatments. The samples were digested with trypsin and labelled with TMT (tandem mass tags, Thermo). The same amount of each labelled sample was mixed, chromatographically separation, and finally subjected to an LC-MS/MS analysis. The peptide mass maps were analysed using Proteome Discoverer^TM^2.2 software (Thermo) and the UniProt database. The significantly different proteins between the two groups were identified with fold change (FC) >1.5 and *p* value (calculated by *t*-test) < 0.05. These differentially expressed proteins were entered as foci into the subsequent clustering analysis, GO analysis and molecular networks generated from the STRING database.

### Statistical Analysis

Data are expressed as mean ± SD, and were analyzed by SPSS 20.0 and Prism 5. The category variables were analyzed with the chi-square test; the continuous variables with *t*-test, ANOVA, or partial correlation. The criterion for statistically significance was set as *p* < 0.05.

## Results

### The Concentrations of Six Active Compounds

The chromatogram of HPLC analysis of DZXI and standard substances solution were shown in Figure 1B. We quantified the concentrations of the six active compounds in DZXI, and the data were as follows: 532.34 μg/ml scutellarin, 226.00 μg/ml 3,4-O-dicaffeoylquinic acid, 110.40 μg/ml 3,5-O-dicaffeoylquinic acid, 179.11 μg/ml erigoster B, 269.81 μg/ml 4,5-O-dicaffeoylquinic acid, and 1,126.04 μg/ml erigeroster.

### Clinical Trial

#### Dengzhanxixin Injection Treatment Improved the Cognitive Functions of Acute Ischemic Stroke Patients

The neuropsychological characteristics of the patients in each group were presented in Table 1. No significant differences were observed between the DZXI group and edaravone group in demographic characteristics, including age, gender, and education levels (Table 1). At baseline, between DZXI group and edaravone group, there were no differences in NIHSS and MMSE tests, but significant differences were observed in some tests, such as ADL, mRS, and TMT A tests. After DZXI and edaravone treatments, the severity of stroke tested with the NIHSS was attenuated and the neurological deficits assessed by ADL and mRS were also alleviated, suggesting that both drugs were effective in AIS treatments. Since cognitive impairments caused by AIS may encompass all types of cognitive disorders, wide-ranging batteries of neuropsychological tests were performed. Compared to the baseline values, the general mental status, executive function, attention, visuospatial ability, memory, and language ability were significantly improved after DZXI treatment. Significant interaction effects between the two treatment groups (DZXI group and edaravone group) and treatment time were observed for the NIHSS, AVLT, and CDT examinations, implying that patients treated with DZXI exhibited better performance on the ALVT and CDT tests than edaravone (Table 1 and Supplementary Figure S2). Therefore, DZXI exerted substantial effects on ameliorating neurological deficits and cognitive impairments in AIS patients, suggesting that DZXI has the ability to improve neurologic function recovery and outcomes of presenting with cognitive impairments.

#### Dengzhanxixin Injection Protected Cerebral Grey Matter in Specific Regions, and This Effect Is Associated With its Effects on Ameliorating Cognitive Deficits in Acute Ischemic Stroke Patients

**FIGURE 2:**
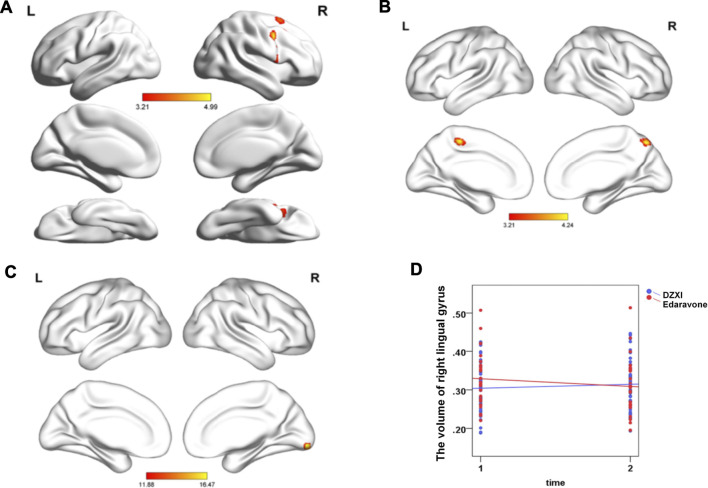
DZXI and edaravone protected the grey matter in specific regions of patients with AIS. Paired *t*-test was used to explore the volume changes between baseline and after 2 weeks of treatment both in the DZXI and edaravone group. A full factorial analysis with age, gender and years of education as covariates was used to explore the interaction between time and groups. After finding the cluster with significant interaction effects between drugs x treatment time, we used the cluster as ROI and extracted the volume value of each subjects. **(A)** The right hemisphere in regions such as the precentral gyrus, central sulcus, superior frontal gyrus, and middle and inferior frontal gyrus were protected by DZXI. **(B)** The left paracentral lobule and right precuneus were protected by edaravone. **(C)** In the comparison of the two time points, the volume of the right lingual gyrus exhibited significant interaction effects between DZXI and edaravone groups. **(D)** DZXI treatment had a better effect on inhibiting the decrease of the volume of the right lingual gyrus, compared with edaravone group. *n* = 47 in DZXI group and 25 in edaravone group. Time 1, baseline; time 2, the end of treatments; blue, DZXI group; red, edaravone group. Each point represents an individual volume of the right lingual gyrus, and lines represent the average volumes.

**FIGURE 3:**
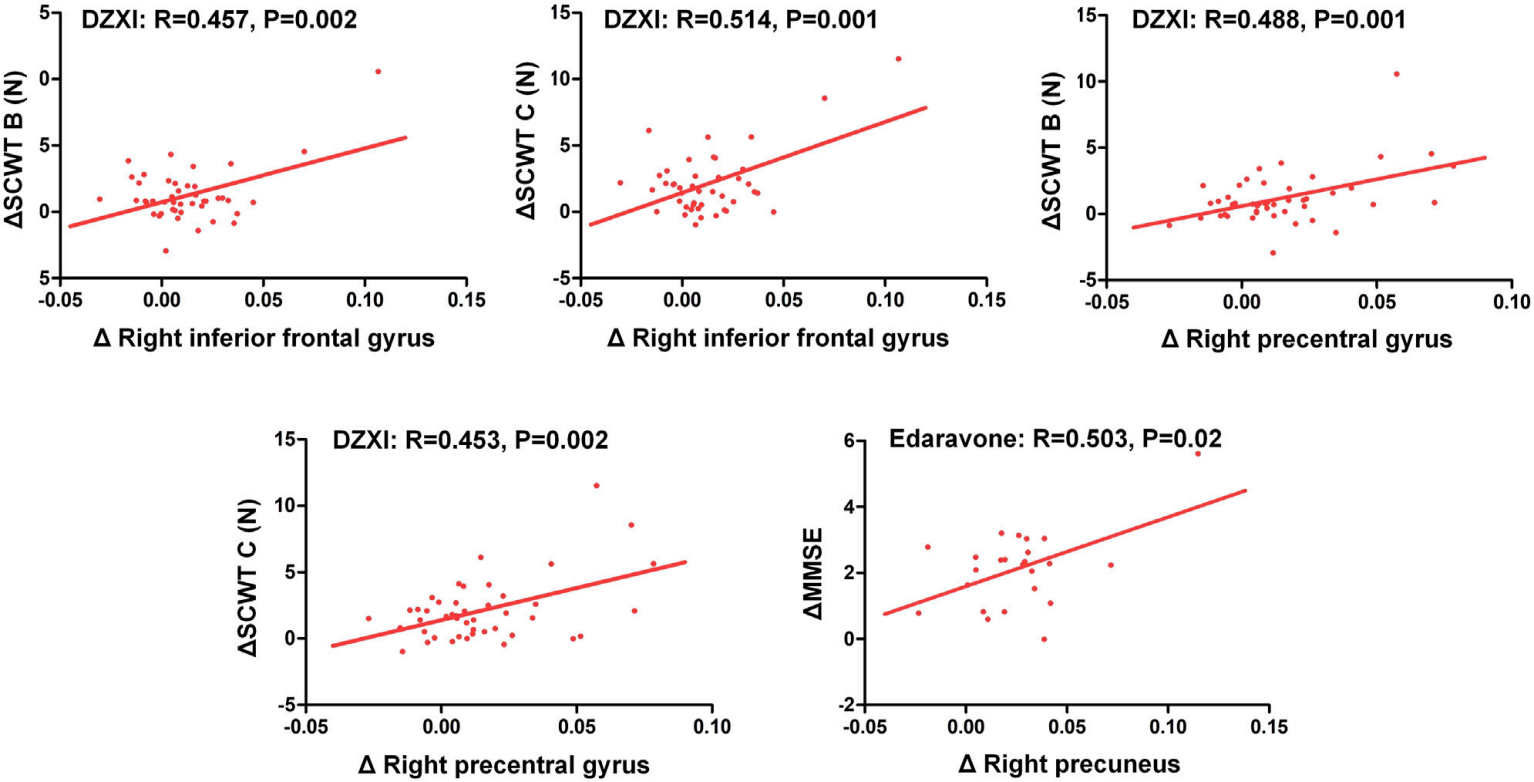
Improvements in the cognitive deficits of AIS patients were associated with increased grey matter volumes in specific regions after treatment with DZXI and edaravone. The association between changes in neuropsychological test scores and that in grey matter volumes of specific regions were analyzed with a partial correlation analysis, with age, gender and years of education serving as the covariates. *n* = 42 in DZXI group and 24 in edaravone group. Δ, the increments in test scores or grey matter volumes at 14th day of treatment from the baseline.

The voxel-based morphometry (VBM) analysis of MRI data revealed improvements in the grey matter volume after the drug treatments (AlphaSim-corrected, *p* < 0.001) (Figure 2; Table 2). Compared to the baseline, the DZXI treatment increased the grey matter volume in the right hemisphere in regions such as the precentral gyrus, the Rolandic operculum (central sulcus), the superior frontal gyrus, the middle frontal gyrus, and the inferior frontal gyrus (opercular part), while the edaravone treatment increased the volume of the left paracentral lobule and the right precuneus. A significant interaction between the two drugs and treatment time was observed in the right lingual gyrus (Figures 2C,D). The specifically altered regions imply that the two drugs may exert different protective effects on the grey matter of AIS patients. Then, the associations between the effects of the drugs on grey matter volumes (ROI based) and that on neuropsychological functions were determined by conducting a partial correlation analysis with age, gender, and years of education as covariates. In DZXI group, the increased amounts in volumes of the right inferior frontal gyrus and the right precentral gyrus were associated with that in scores in SCWT B (N) and SCWT C (N). In edaravone group, this association was only found between the right precuneus and MMSE (Figure 3). The above results imply that the drugs may improve the cognitive impairments through protecting the grey matter, at least partly. It also indicates that treatment to preserve the grey matter in key regions can be a potential therapeutic approach to AIS, especially in cognitive improvements.

**TABLE 2:** The brain regions of grey matter significantly alternated by the drugs treatments in AIS patients.

Brain regions	Cluster size	*x*	*y*	*z*	*t*	*p*
**DZXI group**						
Precentral_R	153	42	2	41	4.99	< 0.001
Rolandic_Oper_R	77	50	3	17	4.31	<0.001
Frontal_Sup_R	53	20	11	63	3.85	<0.001
Frontal_Mid_R	90	32	18	51	3.85	<0.001
Frontal_Inf_Oper_R	69	44	14	8	3.60	<0.001
**Edaravone group**						
Paracentral_Lobule_L	140	−6	−32	53	4.24	<0.001
Precuneus_R	79	6	−68	53	4.14	<0.001
**Interaction**					**F**	
Lingual_R	50	9	−92	−9	16.47	< 0.001

The regions displaying significant alterations after DZXI or edaravone treatment compared to the baseline were shown, analyzed by paired t-test. The *p* values were AlphaSim-corrected. The analysis showed regions with significant interactions. *N* = 47 in DZXI group and 25 in edaravone group.

### Animal Experiment

#### Dengzhanxixin Injection Treatment Protected the Grey Matter and Prevented Infarct Evolution in Middle Cerebral Artery Occlusion Rats

**FIGURE 4:**
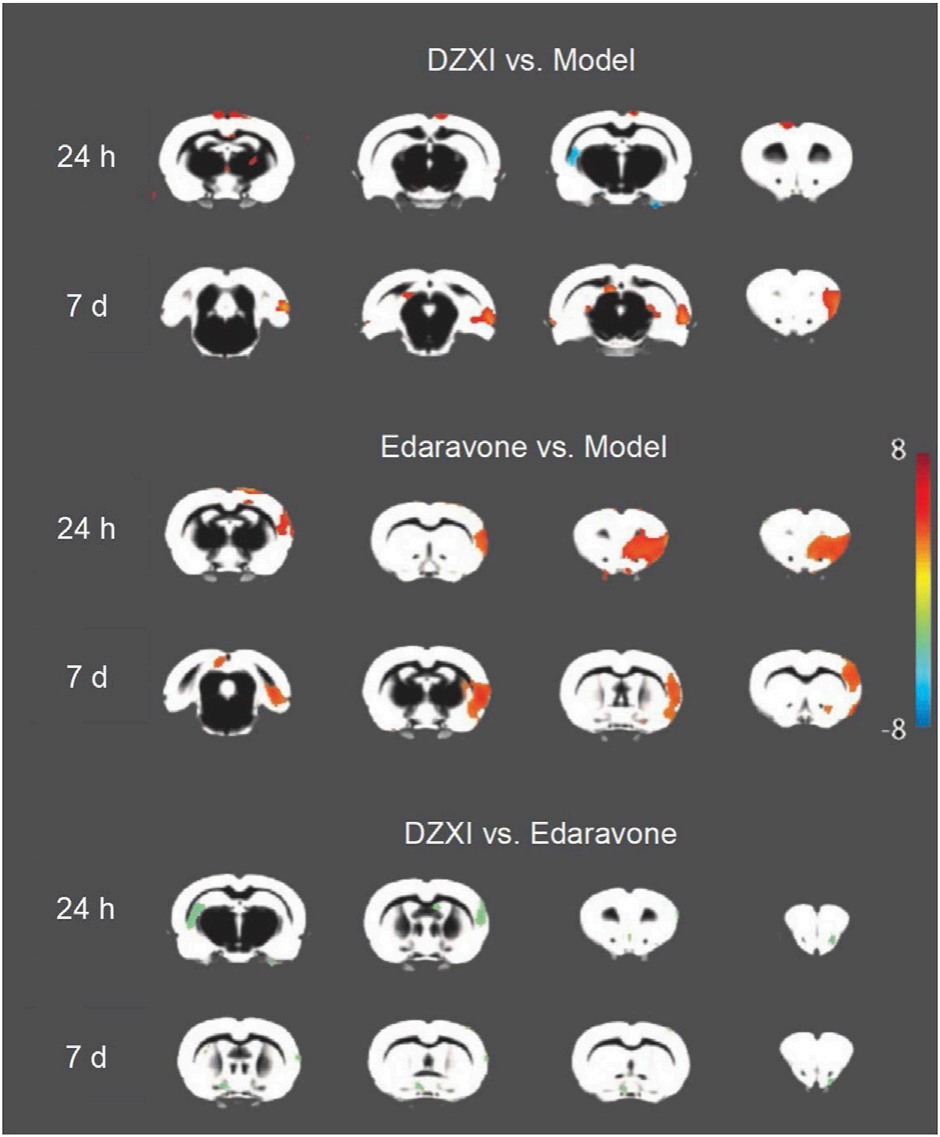
DZXI treatment increased grey matter volumes in specific brain regions of MCAO rats. Due to image quality, one rat was removed from the model group and two rats were removed from the edaravone group. *n* =11 in the model group, 12 in the DZXI group, and 10 in the edaravone group. The changes in grey matter volumes among groups (model group, DZXI group and edaravone group) at 24th h and 7th day of treatments. The brain regions with significant differences in volume among different groups and time pointse were identified by ANOVA analysis performed on the grey matter density map in SPM12. Further pairwise comparisons between groups at each time point were performed using multivariate analysis of variance (MANOVA). The regions with significant changes in volume (Alphasim-corrected *p* < 0.05) were marked in brain maps. DZXI protected some regions in the bilateral neocortex, the right entorhinal cortex, the left perirhinal cortex, the left subiculum and the right frontal association cortex. Edaravone protected some regions in the bilateral neocortex and the olfactory bulb. When compared DZXI and edaravone, a larger volume of the left neocortex was observed in the DZXI group, while larger volumes of the right olfactory bulb, the right neocortex and the left hippocampus were observed in the edaravone group.

We performed an experimental study in the MCAO rats to further elucidate the therapeutic mechanism of DZXI. The intergroup (model group, DZXI group and edaravone group) differences in grey matter volumes were analysed at 24 h and 7 days separately (Figure 4 and Table 3). Compared to the model group, the volume of the bilateral neocortex was larger at 24th h of the treatment in the DZXI group, but the volume of the left hippocampus was smaller, and several regions exhibited larger volumes in the DZXI group at 7th day of the treatment, such as the right entorhinal cortex, the left perirhinal cortex, the left subiculum and the right frontal association cortex. No regions exhibited smaller volumes in the DZXI group at 7th day compared with the MCAO group. In the edaravone group, larger volumes were observed in the bilateral neocortex and the olfactory bulb at 24th h of the treatment and in the bilateral neocortex at 7th day, in comparison to the model group. The comparison between DZXI and edaravone groups at 24th h of the treatment revealed larger volume in the left neocortex in the DZXI group, while larger volume were observed in the right olfactory bulb, the right neocortex and the left hippocampus in the edaravone group. At 7th of the treatment, larger volumes of the right olfactory bulb, the right neocortex and the left hippocampus were still observed in the edaravone group, in comparison to the DZXI group. Notably, in both AIS patients and MCAO model, the frontal cortex was consistently protected by DZXI across all the time points, indicating that targeting the frontal cortex may play a pivotal role in DZXI’s therapy.

**TABLE 3:** The grey matter regions displaying significant volume differences between groups in rats at 24th h and 7th day of treatments.

		Cluster size	x, y, z	*p*	Brain regions
DZXI vs. Model	24 h DZXI> Model	518	14, 68, −54	0.001	Right neocortex
		628	−8, 58, 24	0.001	Left neocortex
		723	−21, 41, 48	0.001	Left neocortex
		19	78, 7, −60	0.005	Right neocortex
	24 h DZXI< Model	278	−55, 19, −45	0.002	Left hippocampus
	7 days DZXI> Model	3219	66, 18, −84	< 0.001	Right entorhinal cortex
		119	−74, 3, −60	0.001	Left perirhinal cortex
		238	−8, 38, −60	0.001	Left subiculum
		2028	47, 10, 33	0.001	Right frontal association cortex
Edaravone vs. Model	24 h Edaravone> Model	7,977	34, 5, 39	< 0.001	Right neocortex1
		1,547	21, 66, −18	< 0.001	Right neocortex2
		36	−5, 59, 27	0.001	Left neocortex
		27	−1, 49, 51	0.002	Left neocortex2
		36	−12, 63, 0	0.002	Left neocortex3
		11	−25, 44, 57	0.002	Left neocortex4
		14	4, 48, 54	0.002	Right neocortex3
		59	11, −16, 30	0.004	Right olfactory bulb
		37	−15, −16, 30	0.005	Left olfactory bulb
	24 h Edaravone< Model	227	−42, 44, 33	0.002	Left neocortex
		34	49, 51, 3	0.003	Right neocortex
	7 days Edaravone> Model	331	−10, 48, −78	0.002	Left neocortex
		16199	59, −10, 9	0.002	Right neocortex
DZXI vs. Edaravone	24 h DZXI> Edaravone	8	−47, 47, 27	0.006	Left neocortex
	24 h DZXI< Edaravone	465	18, 8, 51	< 0.001	Right olfactory bulb
		1,082	64, 26, 12	0.001	Right neocortex
		447	−45, 27, −45	0.001	Left hippocampus
	7 days DZXI< Edaravone	57	19, −4, 45	0.001	Right olfactory bulb
		135	75, 16, −18	0.002	Right neocortex
		105	−36, 25, −15	0.003	Left striatum
		8	66, 5, 9	0.004	Right neocortex2
		29	49, 53, −3	0.006	Right neocortex3

The volumes of grey matter regions in different groups were compared with MANOVA, the *p* values were AlphaSim-corrected. >, larger grey matter volume; <, smaller grey matter volume. *n* = 11 subjects in the model group, 12 in the DZXI group, and 10 in the edaravone group.

**FIGURE 5:**
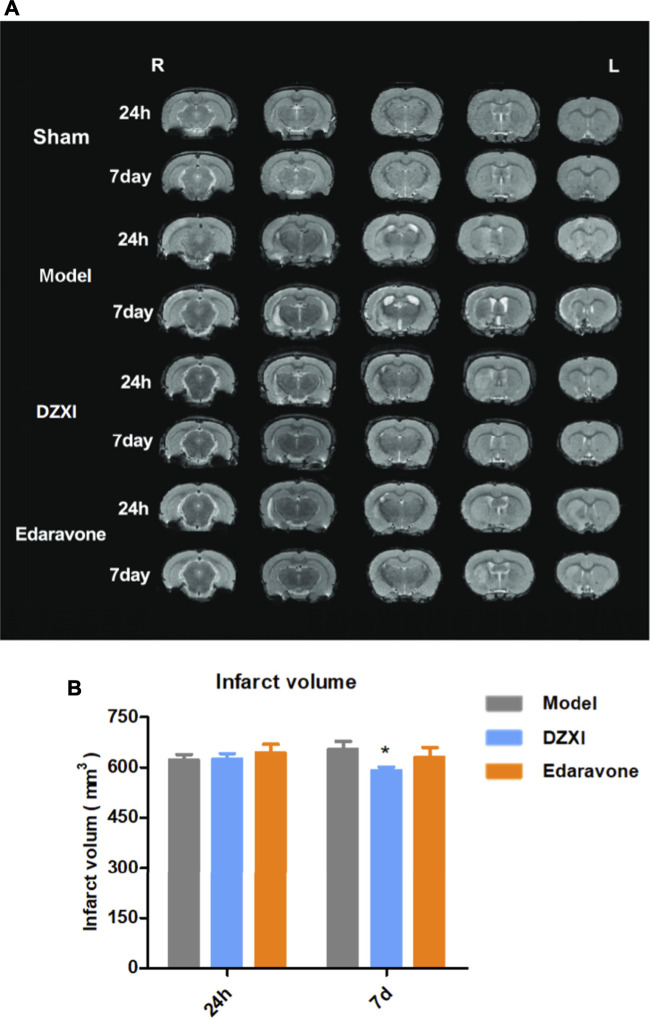
The T2-weighted MRI images and infarct volumes analysis results. **(A)** Representative T2-weighted MRI images of MCAO model, DZXI, and edaravone groups at 24th h and 7th day of treatments. The white regions are infarct regions. **(B)** The infarct volumes of model, DZXI and edaravone groups at 24th h and 7^th^ d of treatments. The differences between groups were analyzed by two-way ANOVA followed by the Bonferroni test. ^∗^ *p* < 0.05, DZXI group vs model group at the same time point.

The dynamical changes of infarct volumes in rat brains were estimated based on T2-weighted MRI. As shown in Figure 5A, ischemic lesions were observed in images of all groups except the sham one, while extensive infarcts were clearly observed in the model group, particularly at 7th day after MCAO, illustrating the aggravation of infarct with time. However, the infract volumes in the DZXI group were significantly reduced at 7th day of the treatment, compared to the model group (Figure 5B). The results were consistent with the findings from the above clinical study and further confirms the effect of DZXI on protecting the cerebral grey matter.

#### Dengzhanxixin Injection Protected Hippocampal and Cortical Neural Cells From Acute Ischemic Damage and This Effect Was Associated With its Preservation on Neuron Mitochondria

**FIGURE 6:**
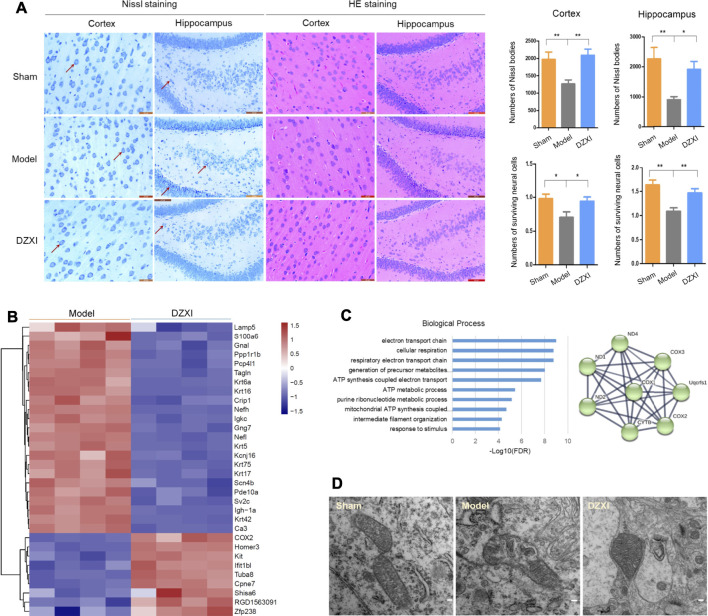
DZXI treatment protected neural cells and neuronal mitochondria. **(A)** The morphology of cortical and hippocampal (dentate gyrus) neurons in DZXI and MCAO model groups were examined using Nissl staining and HE staining. The red arrows indicate the representative Nissl bodies. The numbers of Nissl bodies and surviving neural cells were measured with IPP6 and analyzed with one-way ANOVA followed by the Newman-Keuls test. *n* = 6 in cortex and 5 in hippocampus, ^∗^ *p* < 0.05, ^∗∗^ *p* < 0.01. Bars = 20 μm in the images of the cortex and 50 μm in the images of the hippocampus. **(B)** The differentially expressed proteins between the DZXI group and model group were identified in the proteomic assays, and their clustering figure was assessed by clustering analysis according to their expression levels. The TMT intensities (log_2_Z score transformed) of each protein (rows) in the model group and DZXI group (columns) was indicated on a coloured scale. **(C)** The top 10 biological processes in which the differentially expressed proteins between the model group and DZXI group were involved (left, acquired with GO analysis), and the interaction network of the electron transport chain (right, acquired with STRING). Colored nodes: query proteins and first shell of interactors, edges represent protein-protein associations; line thickness indicates the strength of data support. COX2, cytochrome c oxidase subunit 2; COX3, cytochrome c oxidase subunit 3; ND1, NADH-ubiquinone oxidoreductase chain 1; ND2, NADH-ubiquinone oxidoreductase chain 2; ND4, NADH-ubiquinone oxidoreductase chain 4; CYTB, cytochrome b; Uqcfs1, cytochrome b-c1 complex subunit Rieske. **(D)** The morphology of neuron mitochondria was observed by using transmission electron microscopy at 7th day of treatment. Bar = 100 nm.

Since the results of MRI showed that the grey matter regions altered by DZXI were mainly located in the neocortex and the hippocampus, we focused on these two regions to evaluate the effects of DZXI on neurons of the brain by Nissl and HE staining. At 7th day after MCAO, the number of Nissl bodies significantly decreased in the cortex and the hippocampus in the MCAO model group compared with the sham group, indicating that ischemia resulted in severe neuron damage. Compared to the MCAO model group, the number of Nissl bodies significantly increased in the DZXI group, indicating that DZXI treatment alleviated this neuronal injury. Similarly, compared with the sham group, the number of surviving neural cells evaluated with HE staining also obviously decreased in the cortex and the hippocampus in the MCAO model group. Compared with the MCAO model group, the number of surviving neural cells significantly increased in the DZXI group. The neural cells in the DZXI group exhibited improved morphological features, such as a more orderly arrangement and more intact cell structures (Figure 6A). These results illustrate that the DZXI treatment protected the brain neural cells from ischemic injury, which may be associated with its ameliorative effects on ischemic stroke.

In the proteomic study, we identified 32 differentially expressed proteins between the DZXI and model groups at 7th d (Supplementary Table S1), and the clustering analysis of these proteins was shown in Figure 6B. The GO analysis showed that the mitochondrial electron transport chain has the closest association with proteins modulated by DZXI (Figure 6C). The results indicated that mitochondria may be a main protective target of DZXI. The protection of DZXI on mitochondria was also supported by the results of transmission electron microscopy. At 7th day of the treatment, the sham group had many neurons mitochondria with completed morphology, including an intact mitochondrial outer membrane, complete and clear mitochondrion cristae, and a typical homogeneous staining pattern of the matrix. While the MCAO model group had many injured mitochondria in neurons of the penumbra regions. The mitochondria seriously swelled, the neurons outer mitochondrial membranes were ruptured, the mitochondrial cristae decreased or disappeared and became incomplete and unclear, with signifificant differences from the sham group. Compared with the MCAO model group, quite a number of sound mitochondria in the DZXI group presented in neurons of the penumbra regions. The mitochondria presented a relatively completed morphology, including regular shape, relatively intact outer membranes, and increased and clear mitochondrial cristae (Figure 6D). These results suggest that DZXI treatment plays a protective role by maintaining the stability of neuron mitochondrial morphology during ischemic stroke.

## Discussion

In the past 2–3 decades years, the overall burden of ischemic stroke has been rising due to population aging and the high stroke cases with enormous daily life risk factors which are hard to control (Wang W. et al., 2017; Guan et al., 2017). The currently available treatment for AIS stroke includes rt-PA and mechanical thrombectomy. However, they also elicit severe reperfusion injuries and are not yet suitable for wide use. These limitations largely lead to the presence of serious sequelae in stroke survivors, which are becoming more and more prevalent as the stroke death rate is continuously decreasing (Heit et al., 2018; Campbell and Khatri, 2020). The pathological mechanisms of AIS involves hypoxia, free radicals generation, vascular damage, cell apoptosis, inflammation, and other events leading to neural cells damage and functional impairment associated with the brain regions in the ischemic penumbra area, resulting in infarction area enlargement (Xu et al., 2021). The drug therapy reduced the disability rate and attenuated cognitive decline in the acute phase of ischemic stroke, resulting in better clinical outcomes for patients with AIS.

According to previous studies, AIS patients with NIHSS scores of 3–10 points are with greatest long-term risk of dementia, and in general, a score of less than 24 points on the MMSE at baseline is one of the strongest predictors of more severe stroke-related neurological deficits (Dichgans, 2019). Therefore, the patients enrolled in our study had a high risk for subsequent severe functional deficits. We found that DZXI treatment prevented the aggravation of stroke injury and improved the recovery from the neurological impairments, thus enhancing the abilities of daily living. Our results support the theory that the drug intervention can prevent cognitive decline in AIS patients. Reassuringly, DZXI exerted more beneficial effects on visuospatial function and episodic memory than edaravone, indicating that DZXI might be more effective on improving cognitive impairment than edaravone. However, cognitive impairments occur weeks or even months after ischemic stroke (Mijajlovic et al., 2017), and the cognitive time courses vary (Wang et al., 2012). Therefore, the cognitive improvement benefits of DZXI should be further confirmed in large, rigorous and long-term trials.

Determinants of cognitive impairments after ischemic stroke comprise infarct features such as the location and size, white matter changes and extent of grey matter atrophy (Jouvent et al., 2016). One of the most frequently reported neuromaging biomarkers for the acute stroke is progressive cerebral atrophy, including decreases in the regional grey matter volume and cortical thickness (Yatawara et al., 2018). Several previous studies indicated that the infarction location of AIS is associated with neurological and cognitive impairments. These brain regions include basal ganglia, thalamus, brainstem, insula, frontal cortex, temporal cortex, hippocampus and cingulum cortex, precuneus, precentral gyrus, and paracentral lobule (Galovic et al., 2013; Cheng et al., 2014; Wu et al., 2015; Zhu et al., 2019). In the present study, DZXI increased the grey matter volume of the right precentral gyrus, the right central sulcus, the right superior frontal gyrus, the right middle frontal gyrus, and the right inferior frontal gyrus in patients with AIS. These brain regions are associated with cognitive function since several distinct cognitive and behavioral processes are reportedly mediated by them (Henri-Bhargava et al., 2018; Parnaudeau et al., 2018; Zanto and Gazzaley, 2019). Previous studies showed functional connectivity decrease in the left paracentral lobule, left middle frontal gyrus, and the right middle frontal gyrus in patients with vascular cognitive impairment (Sun et al., 2011). In this study, the edaravone treatment increased the volume of the left paracentral lobule and the right precuneus. The grey matter volume in specific regions in both human (stroke patients) and model animals was successfully restored by DZXI treatment. The grey matter regions that were alleviated by DZXI, such as the frontal cortex (observed in patients and rats) and the hippocampus (only observed in rats), are all key regions involved in cognitive function. The differences in protected grey matter regions between patients and rats may be attributed to different time course (length, for example) of the treatments. The protective effects of DZXI on the central sulcus should also be highlighted, because the morphological variation of the central sulcus is associated with functional recovery in patients with small subcortical ischemic stroke (Jouvent et al., 2016).

In addition, the difference in changes in grey matter regions between the DZXI and edaravone groups indicates that the two drugs might exert their protective effects through different mechanisms.

Importantly, the volumes of regional cerebral grey matter were associated with cognitive outcomes after stroke, and this finding is consistent with previous reports. In a prospective study, ischemia resulted in a significant reduction in the global grey matter volume and focal structural atrophy, which further led to a proportional decrease in cognitive performance (Bivard et al., 2018). According to another report, the decreased grey matter volume may also explain the long-term motor deficits in patients with stroke (Yang et al., 2015). Notably, the better effect of DZXI than edaravone on improving visuospatial function assessed using the CDT was consistent with its better effect on increasing the grey matter volume in the lingual gyrus, which is responsible for visual function and participates in the processing of visual memory. Hence, the findings of grey matter alterations might partly explain the different performances of the patients treated with the two drugs on a series of neuropsychological tests. The substantial and rapid loss of neurons is the core pathological mechanism that leads to acute-stage injuries, including paralysis, coma and even death, and is also the direct cause of brain atrophy and cognitive impairments in AIS (Saver, 2006). Our results support the therapeutic notion which believes targeting neurons as a potentially effective strategy for AIS.

The ischemic penumbra is located around the infarction and is viewed as an “at risk” region (Egeland, 2015). Actually, the neuronal cells in this zone undergo less grievous impairments and remain viable for hours after vessel occlusion (Lo, 2008b; El-Koussy et al., 2014). Based on these characteristics, the ischemic penumbra is relatively easy to salvage and has a higher therapeutic window serving as an appropriate intervention target (Hakim, 1998; Baron, 2018). However, unless blood perfusion is improved or cells become more resistant to injury, the ischemic penumbra is rarely to maintain basal activities and will die through a series of complex events involving excitotoxicity, oxidative stress, programmed cell death, and inflammation (Lo, 2008a; Heiss, 2012). Combining the results of morphological examinations of neuronal cells with the infarct evaluation in our study, we inferred that the protective effect of DZXI on neural cells, particularly on those in the penumbra regions, may underlie its ability to alleviate ischemia-induced grey matter damage. Thus, the neuroprotective effect of DZXI supports the therapeutic strategy that proposes interrupting the transfer to infarct by rendering viable brain in the penumbra more resistant to ischemia (Moskowitz et al., 2010).

The results of the proteomic analysis and transmission electron microscopy examination revealed the protective effect of DZXI on mitochondria, which might allow the neural cells to become more resistant to ischemic injury. Mitochondrial dysfunction has been observed in AIS (Hakim, 1998) and in the course of Alzheimer’s disease progression (Xie et al., 2018). The levels of mitochondrial electron transport chain subunits in neurons have been reported to be decreased in the posterior cingulate cortex (PCC) and other brain regions in AD patients (Zhou et al., 2017). The mitochondrial cytochrome oxidase levels have also been found to be reduced in PCC neurons in cognitively unimpaired young-adult APOE4 gene carriers, those at greater risk for developing AD (Heiss, 2012). These results indicate that changes in mitochondrial function may contribute to cognitive decline. Importantly, recent studies have revealed mitochondria also exert one of the main endogenous protective effects in AIS (Anzell et al., 2018; Liu et al., 2018). The brain can be “trained” to resist or tolerate ischemic stroke injury, and the mitochondria are a major target of this training (Jin et al., 2016). Approaches that maintain mitochondrial activities would be an extremely promising strategy for the early prevention of cognitive decline and improving cognitive stability (Wang et al., 2014). In our study, DZXI treatment modulated mitochondrial electron transport chain process, significantly increased the levels of cytochrome c oxidase subunit 2 (COX2), and protected the mitochondrial stability in neural cells. With all these positive and interesting findings, we noted that the direct protein target and the biological process activated by the DZXI treatment were not elucidated in the present study; thus, future studies aiming to further explore the molecular mechanism underlying the modulatory effects of DZXI on AIS are necessary.

Our results revealed the effects of DZXI on improving cognition and protecting cerebral grey matter in AIS patients presenting with cognitive impairments. Recent pharmacological experiments revealed that the main components of DZXI, have multiple beneficial effects (Wang et al., 2012; Wang J. et al., 2017; Wang and Ma, 2018). Particularly, scutellarin has been reported to have the ability to regulate glutamatergic and GABAergic neuron synapses (Sheng et al., 2020) and prevent cognitive decline in AD transgenic mice (Zhang et al., 2020). These findings suggest that scutellarin and caffeic acid ester may build the material foundation for DZXI therapeutic effect on cognitive impairment in AIS.

The safety of DZXI for AIS has been shown in a meta-analysis of randomized controlled trials and systematic review. Twenty-five RCTs with 2,498 participants were included. Thirteen trials (52%) reported the outcome of adverse events including slight ecchymosis at the injection site, mild blood pressure decreasing, slightly abnormal coagulation function, and mild fever or skin rash, which all relieved within the treatment duration without special management, but no serious adverse events were reported (Li et al., 2017). No adverse events occurred in this study. According to medicine operation instructions, the side effects include allergic reactions (flushing, itchy skin, rash, dyspnea, feel suffocated, palpitation, blood pressure reduce, anaphylactic shock) and chills, fever, high fever, fatigue, sweating, nausea, vomiting, Chest discomfort, dizziness, headache.

Placebo-controlled clinical trials are difficult to conduct because of the high disability rate caused by AIS. In this study, edaravone was used as positive control drug. Earavone is widely used to treat ischemic injury to the nervous system and is an effective free radical scavenger recommended for AIS treatment by Chinese and Japanese stroke care guideline (Xu et al., 2019). Edaravone has been shown to inhibit lipid peroxidation and vascular endothelial cell injury, and to ameliorate brain edema, tissue injury, delayed neuronal death and neurological deficits. A multicenter, randomized, placebo-controlled, double-blind clinical trial of edaravone was performed in 2001. Among 250 AIS patients treated within 72 h of onset, a significant improvement in functional outcome was observed in the edaravone group (Kitagawa, 2006). The reduction of intracellular ROS and suppression of superoxide in neutrophils provided a potential explanation for the clinical efficacy of edaravone in patients with ischemic brain attack (Aizawa et al., 2006). In this study, DZXI exerted better effects than edaravone in some neuropsychological tests in AIS patients, which suggests indirectly that DZXI contributes to the clinical recovery of AIS patients.

There exists evidence that edaravone, scutellarin, and caffeic acid (5-lipoxygenase inhibitor) contributed to cellular survive through protecting mitochondrial integrity and reducing ROS in cell model. For *in vitro* experiment of glucose deprivation (OGD)-reperfusion induced PC12 cells model, edaravone preserved the normal ultrastructure of neuronal cells’ mitochondria by inactivating 5-lipoxygenase pathway and reduced ROS, and caffeic acid prevented neuronal cells from undergoing apoptosis, significantly decreased the production of arachidonic acid by lipoxygenase metabolism, maintained the mitochondrial ultrastructure integrity of neuronal cells, enhanced mitochondrial membrane potential (MMP), and decreased ROS (Song et al., 2018). Previous studies have indicated that scutellarin has antioxidation, antioxidative stress, anti-ischemic, and anti-inflammatory effects (Wang and Ma, 2018; Wu and Jia, 2019). Scutellarin ameliorated high glucose-induced vascular endothelial cells injury by activating PINK1/Parkin signal pathway-mediated mitophagy. It improved the overload of ROS, superoxide dismutase (SOD) activity and SOD2 protein expression, and reversed the collapse of MMP in high glucose-induced vascular endothelial cells model (Xi et al., 2021). Scutellarin may have a similar mechanism as edaravone. Therefore, the complex drug synergy interactions and similar mechanism deserve further investigation by means of pharmacological methods in the future. Based on current experimental data, the direct target is indefinite and deserves further study. The cell preference of the compound is also unclear due to lack of nerve cells experiments *in vitro*. Previous studies have indicated that scutellarin pre-treatment increase rat neurons viability and suppressed malondialdehyde level in early stages of neuron damage induced by hydrogen peroxide (Liu et al., 2005).

Several limitations exist in the current study and should be addressed in the future. They include a short duration evaluation, drug multicomponent actions which made the direct target not clearly identified, and lack of edaravone molecular mechanisms for reasons elaborated more below. Future longitudinal studies are needed which would be essential to confirm our findings in post stroke cognitive impairment. DZXI includes six compounds, each and optimal combination of which deserve further investigation in animal model to illustrate potential effects and possible mechanism. Edaravone is widely used to treat ischemic injury to the nervous system and is an effective free radical scavenger recommended for AIS treatment by Chinese and Japanese stroke care guidelines. As shown in proteomic results, the therapeutic effects of DZXI are multi-target and multi-pathway synergy effect of six chemical components in AIS. The properties of chemical components and molecular mechanism of edaravone and DZXI are obviously different. Therefore, edaravone was only used as a positive control drug of therapeutic effect and its molecular mechanisms was not the focuse of this study. Rat brain tissues around the ischemic core of edaravone group were not detected by TMT proteomic analysis in animal experiment. We will investigate the protective effects of DZXI and six compounds on mitochondrial integrity, respiratory chain, and molecular mechanisms *via* neural cell model in the future.

In summary, our data suggested that DZXI intervention can ameliorate certain neurological deficits and cognitive impairments in patients with AIS. Its therapeutic effects were the protection on cerebral grey matter volumes in several relevant brain regions, the neurons and the neuron mitochondria. These results demonstrate that DZXI treatment has a potential to become a valuable supplementation or alternative in ischemic stroke therapy.

## Data Availability

The original contributions presented in the study are publicly available. This data can be found here: http://proteomecentral.proteomexchange.org/cgi/GetDataset?ID=PXD026918.
