# Supplementary material for: Dengzhanxixin Injection Ameliorates Cognitive Impairment Through a Neuroprotective Mechanism Based on Mitochondrial Preservation in Patients With Acute Ischemic Stroke
Source: Front Pharmacol. 2021 Aug 30;12:712436. doi: 10.3389/fphar.2021.712436 (PMC8435665; doi:10.3389/fphar.2021.712436)
Supplement: Supplementary file 2 [file DataSheet1.docx]

**Supplementary Figures**


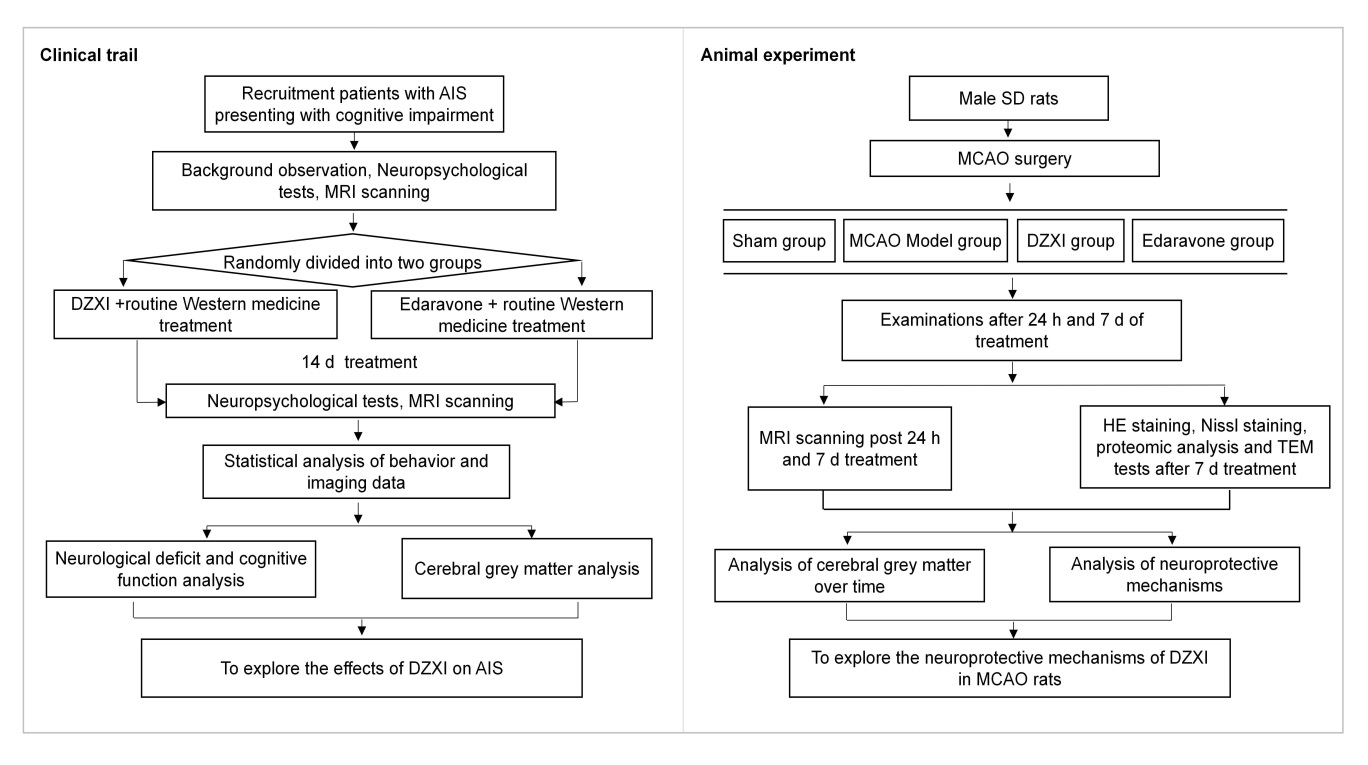


**Supplementary Figure S1.** The procedures of the clinical trial and animal experiment.

**
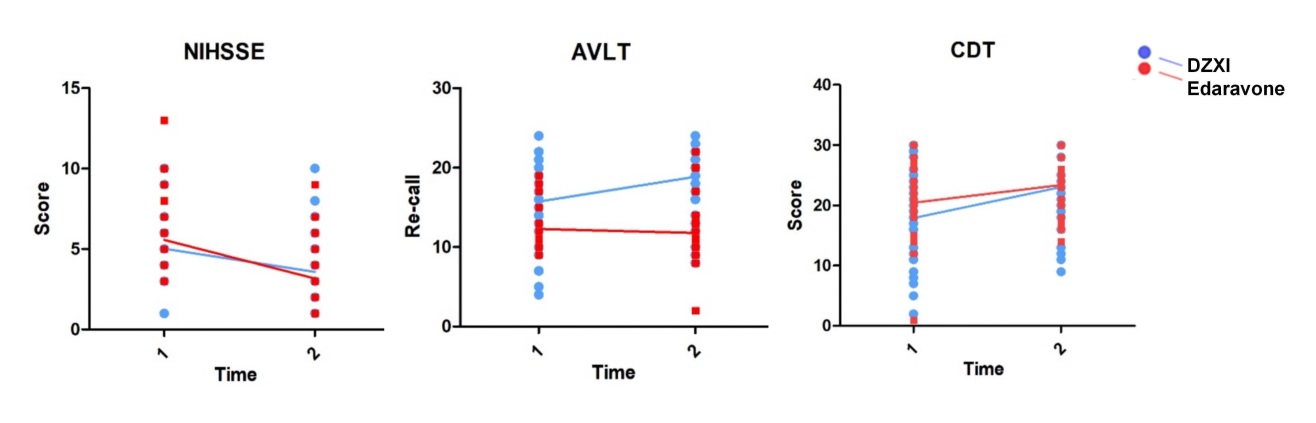
**

**Supplementary Figure S2.** DZXI and edaravone exerted different performance in the NIHSS, AVLT and CDT. The significant interaction effects between treatment groups (DZXI and edaravone) and treatment time were shown in these neuropsychological tests as indicated by two-way repeated measures ANOVA analysis. Time 1, baseline; time 2, the end of the treatment; blue, DZXI group; red, edaravone group. Each point represents an individual’s score on each neuropsychological test, and lines links the average at the two points.
